# γ-tocotrienol regulates gastric cancer by targeting notch signaling pathway

**DOI:** 10.1186/s41065-023-00277-w

**Published:** 2023-04-13

**Authors:** Ling Xie, Juan Yan

**Affiliations:** 1 Key Laboratory of Spine and Spinal Cord Injury Repair and Regeneration of Ministry of Education, Orthopaedic Department of Tongji Hospital, School of Life Sciences and Technology, Tongji University, Shanghai, 200092 China; 2 College of Food Science and Technology, Shanghai Ocean University, Shanghai, 201306 China

**Keywords:** γ-tocotrienol, Gastric cancer, Mitochondria, Oxidative phosphorylation, Notch signaling pathway

## Abstract

**Background:**

Gastric cancer is a common cause of death from cancer and an important global health care issue. Consequently, there is an urgent need to find new drugs and therapeutic targets for the treatment of gastric cancer. Recent studies have shown that tocotrienols (T3) have significant anticancer ability in cancer cell lines. Our previous study found that γ-tocotrienol (γ-T3) induced apoptosis in gastric cancer cells. We further explored the possible mechanisms of γ-T3 therapy for gastric cancer.

**Methods:**

In this study, we treated gastric cancer cells with γ-T3, collect and deposit the cells. γ-T3-treated gastric cancer cells group and untreated group were subjected to RNA-seq assay, and analysis of sequencing results.

**Results:**

Consistent with our previous findings, the results suggest that γ-T3 can inhibit mitochondrial complexes and oxidative phosphorylation. Analysis reveals that γ-T3 has altered mRNA and ncRNA in gastric cancer cells. Significantly altered signaling pathways after γ-T3 treatment were enriched for human papillomavirus infection (HPV) pathway and notch signaling pathway. The same significantly down-regulated genes notch1 and notch2 were present in both pathways in γ-T3-treated gastric cancer cells compared to controls.

**Conclusions:**

It is indicated that γ-T3 may cure gastric cancer by inhibiting the notch signaling pathway. To provide a new and powerful basis for the clinical treatment of gastric cancer.

## Introduction

Cancer is a major public health problem worldwide. In countries around the world, cancer is a leading cause of death and poses a significant barrier to increasing life expectancy [12]. According to World Health Organization (WHO) estimates, cancer is the top four cause of death by age 70 [55]. Collectively, this reflects the rapidly increasing burden of cancer morbidity and mortality worldwide [40]. Gastric cancer is the second most common cause of cancer deaths worldwide [26]. Gastric cancer is caused by many factors, such as smoking and a diet high in nitrates and nitrites or infection with helicobacter pylori. It also includes some non-modifiable factors, such as age, gender and race [26]. The treatment of early gastric cancer is mainly endoscopic resection. Patients with advanced gastric cancer require chemotherapy. Some of the currently approved targeted therapies for the treatment of gastric cancer include trastuzumab, ramilumab and nivolumab or pembrolizumab [53]. However, the treatment methods for gastric cancer need to be advanced so as to bring benefits to patients.

More and more studies are focusing on finding more effective anticancer drugs from plant reservoirs [2, 15, 36]. T3 are naturally occurring compounds of the vitamin E family and have been reported to be present in the seeds of plants [51]. Studies have reported the role of T3 in various types of cancer. T3 exert their anticancer effects in multiple ways, including inhibition of angiogenesis, promotion of apoptosis and metastasis [16, 37, 47]. T3 is divided into four isomers, including α-T3; β-T3; γ-T3; and δ-T3 [4]. Of which, γ-T3 and δ-T3 exhibited stronger anticancer activity [5].

Studies have confirmed that T3 exerts anticancer activity mainly by inhibiting the transcription factors NF-κB and STAT3 and their regulatory gene products [34]. Furthermore, it was found that in pancreatic cancer cell lines, γ-T3 and δ-T3 effectively inhibited the activation of Akt to induce apoptosis in pancreatic cancer cells [52]. Studies have reported that γ-T3 can increase the production of mitochondrial reactive oxygen species (ROS) to induce apoptosis in human T-cell lymphoma Jurkat cells [62]. In addition, one study reported that γ-T3 can activate both ER stress-mediated apoptosis and autophagy to enhance cell death in breast cancer cells [57]. Therefore, γ-T3 has great research value in the treatment of gastric cancer.

The notch signaling pathway plays an important role in immune cell generation [17, 39]. Dysregulation of the notch signaling pathway leads to multiple pathophysiologies in cancer diseases. Notch signaling pathway was found to be involved in cell proliferation and apoptosis [44], and dysfunctional notch signaling was reported to induce gastric cancer [63]. It was found that the expression levels of notch1/2/3 were higher in gastric cancer tissues compared to normal tissues. It is believed that notch1/2/3/4 may be a potential target for precision treatment of gastric cancer [23].

Our previous study showed that γ-T3 was able to induce apoptosis in gastric cancer cells [59]. To further investigate the mechanism of γ-T3-induced apoptosis in gastric cancer, we performed RNA-seq assay on γ-T3-treated MKN-45 cells. Firstly, analysis of RNA-seq results from γ-T3-treated gastric cancer cells and untreated cells revealed significant differences in mRNA and ncRNA between the two groups. There were 669 up-regulated genes and 320 down-regulated genes in mRNA in the γ-T3-treated group. Additionally, there were 95 up-regulated genes and 42 down-regulated genes in ncRNA in the γ-T3-treated group. We analyzed mRNAs with down-regulated gene expression using kyoto encyclopedia of genes and genomes (KEGG) and found common genes notch1 and notch2 in the gene-enriched human HPV and notch signaling pathway. These results suggest that γ-T3 induces apoptosis in gastric cancer cells by downregulating notch1 and notch2 gene expression in the notch signaling pathway. To establish a basis for the use of γ-T3 as a clinical target for the treatment of gastric cancer.

## Materials and methods

### Reagents

γ-T3 (≥ 97%) was purchased from Hygeia Industries Inc (USA). γ-T3 was dissolved in ethanol (95%) to make a 100 mM solution and stored at -20 °C. RPMI-1640 medium, fetal bovine serum, 0.25% EDTA, and penicillin/streptomycin were purchased from Gibco (Grand Island, NY, USA).

### Cell culture

The human gastric cancer cell line MKN-45 (obtained from the Cancer Institute of the Chinese Academy of Medical Science) were cultured in RPMI 1640 containing 10% fetal bovine serum and 1% penicillin/streptomycin at 37 °C in a humidified incubator with 5% CO_2_. MKN-45 cells were seeded into 96-well plates at 5000 cells per well and allowed to adhere overnight. MKN-45 cells were treated with γ-T3 (30umol/L) or not for 24 h. Cells were collected and total cellular RNA was isolated using TRIzol reagent (Molecular Research Center, USA).

### RNA-seq

RNA extraction and quality control and RNA-seq library construction and quality control experiments were performed by Shanghai Rongxiang Biotechnology Co., Ltd. The constructed RNA-seq libraries were sequenced by illumina sequencer. Quality control and analysis of the sequenced data were performed by Shanghai Rongxiang Biotechnology Co., Ltd. Gene expression was expressed by FragmentsPer Kilobase per Million (FPKM), and differential gene expression analysis was performed using Deseq2 to compare the treatment and control groups, and genes with |log2FoldChange|≥ log21.5 and padj < 0.05 were selected as differentially expressed genes screening criteria to obtain up down-regulated genes [35]. Differences between the treatment and vehicle groups were assessed using multivariate linear models. The Bonferroni multiple correction method was used to correct for false discovery errors. *p*-value < 0.05 was set as the threshold of significance for screening out differentially expressed genes (DEGs). DEGs were then used for volcano plot, heat map and functional analysis. ClusterProfiler was used to evaluate biological pathways [65] and we performed gene ontology (GO) [9], KEGG pathway [30] and gene set enrichment analysis (GSEA) [29].

### Statistical analysis

Sequencing data were processed using DEseq2 software. mRNAs and ncRNAs were defined as differentially expressed when |log2FoldChange|≥ log21.5 and padj < 0.05. *p*values < 0.05 were considered statistically significant.

## Results

### RNA-seq assay of γ-T3-treated gastric cancer cells

**Fig. 1:**
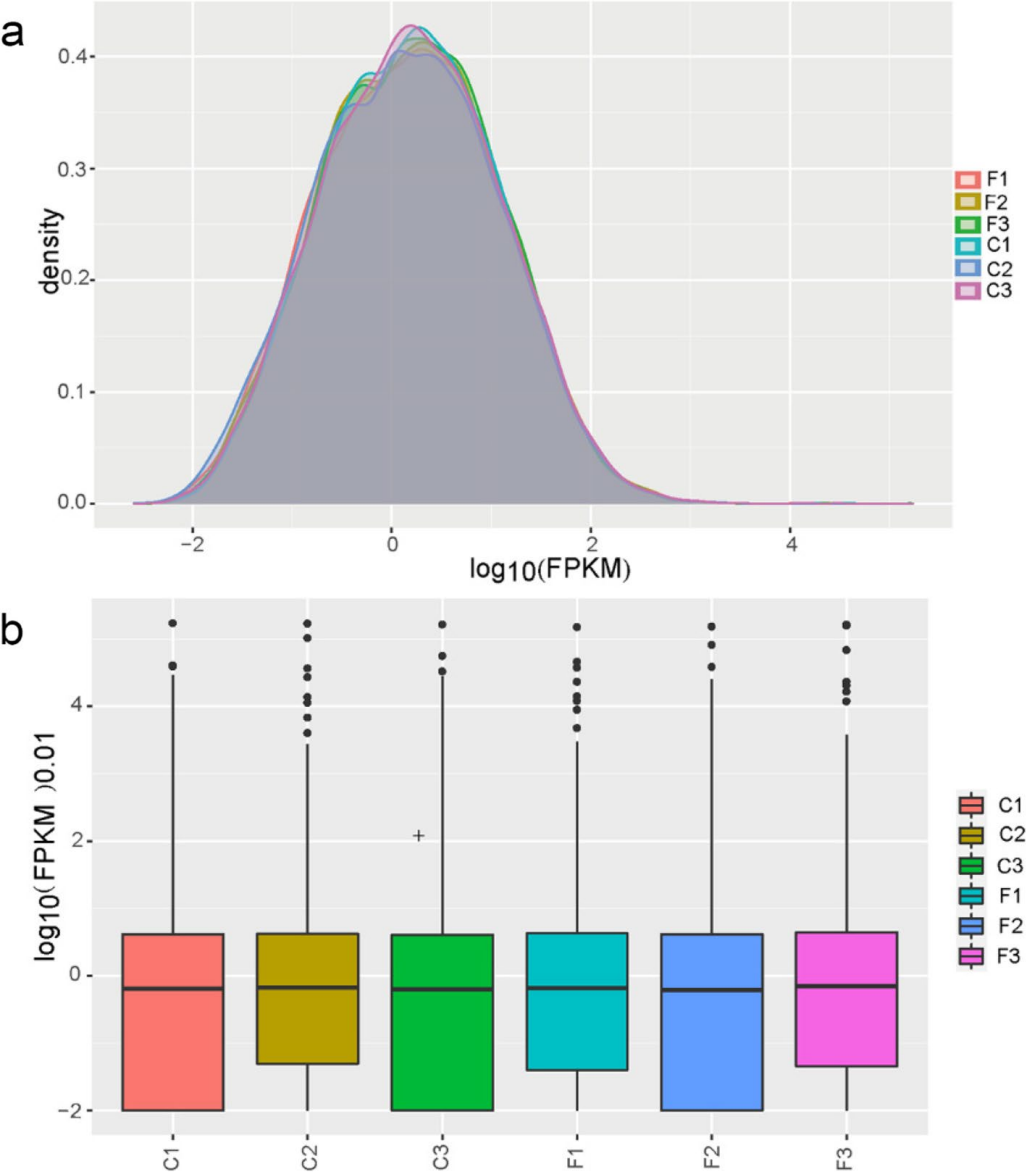
RNA-seq assay of γ-T3-treated gastric cancer cells. MKN-45 cells were incubated for 24 h with γ-T3 (30umol/L). **a** Expression density plots of γ-T3-treated and control samples. **b** Expression distribution boxplot of γ-T3-treated and control samples. F: γ-T3-treated group; C: control group. *n* = 3

To investigate the mechanism of the role of γ-T3 in regulating gastric cancer. We performed RNA-seq assay on γ-T3-treated MKN-45 cells and untreated group cells, and analyzed the data for both groups of results. Based on the gene expression of all samples, an expression density plot of the samples was obtained (Fig. 1a), and the overall distribution trend of the expression of the samples was viewed using the distribution boxplot of the expression of all samples (Fig. 1b). The results showed differentially altered genes in the γ-T3-treated group compared to the control group.

### γ-T3 affects gastric cancer cells

**Fig. 2:**
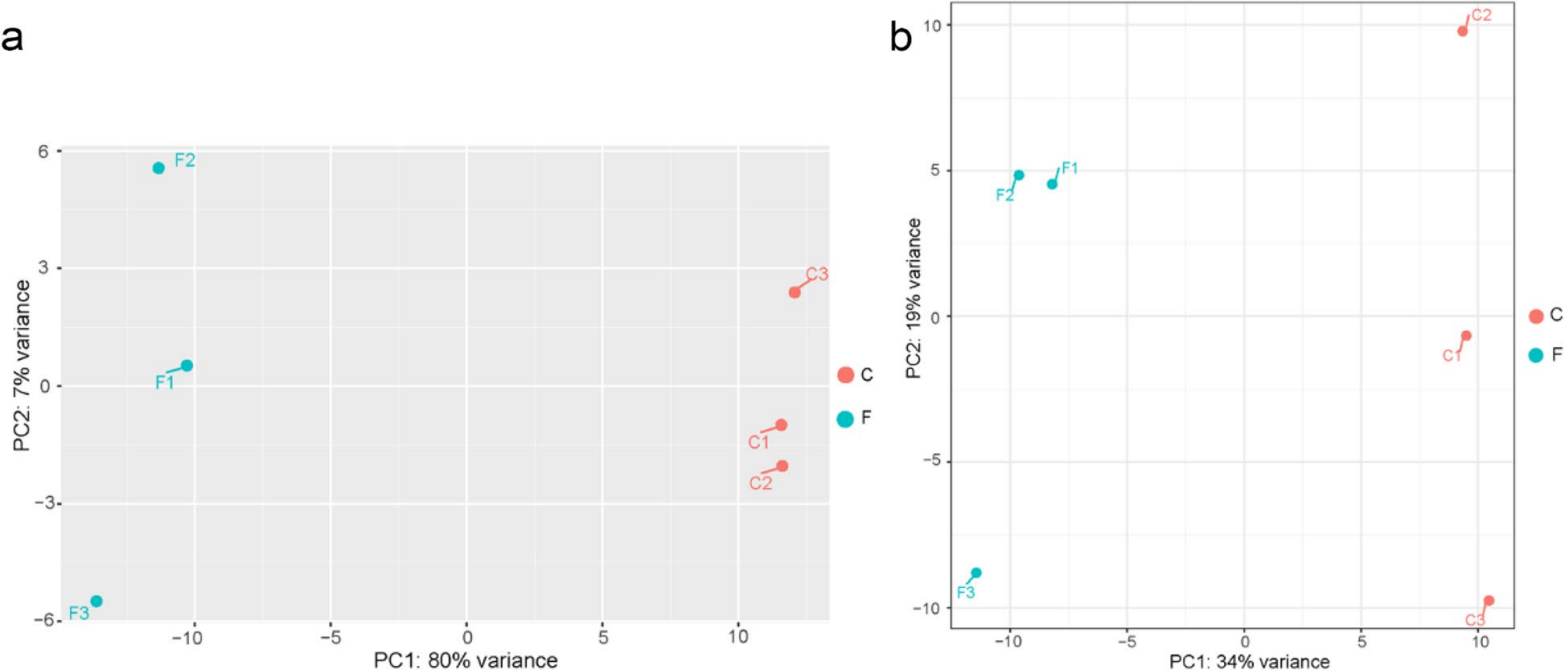
Changes in γ-T3-treated gastric cancer cells. **a** Principal component analysis of mRNA. **b** Principal component analysis of ncRNA. F: γ-T3-treated group; C: control group. *n* = 3

By performing principal component analysis (PCA) on the mRNA data of each sample detected by RNA-seq in the γ-T3-treated and untreated groups, the results showed the feasibility of three replicates for both experimental groups and differences between the two experimental groups (Fig. 2a). Meanwhile, we performed PCA on the ncRNA data of each sample from the RNA-seq assay of the γ-T3-treated and untreated groups, and the results similarly showed the feasibility of three replicates of the two experimental groups and the difference between the two experimental groups (Fig. 2b). The results showed that there was a significant difference between the γ-T3-treated group and the untreated group, and the treatment of γ-T3 had an effect on gastric cancer cells.

### γ-T3 alters gene expression in gastric cancer cells

**Fig. 3:**
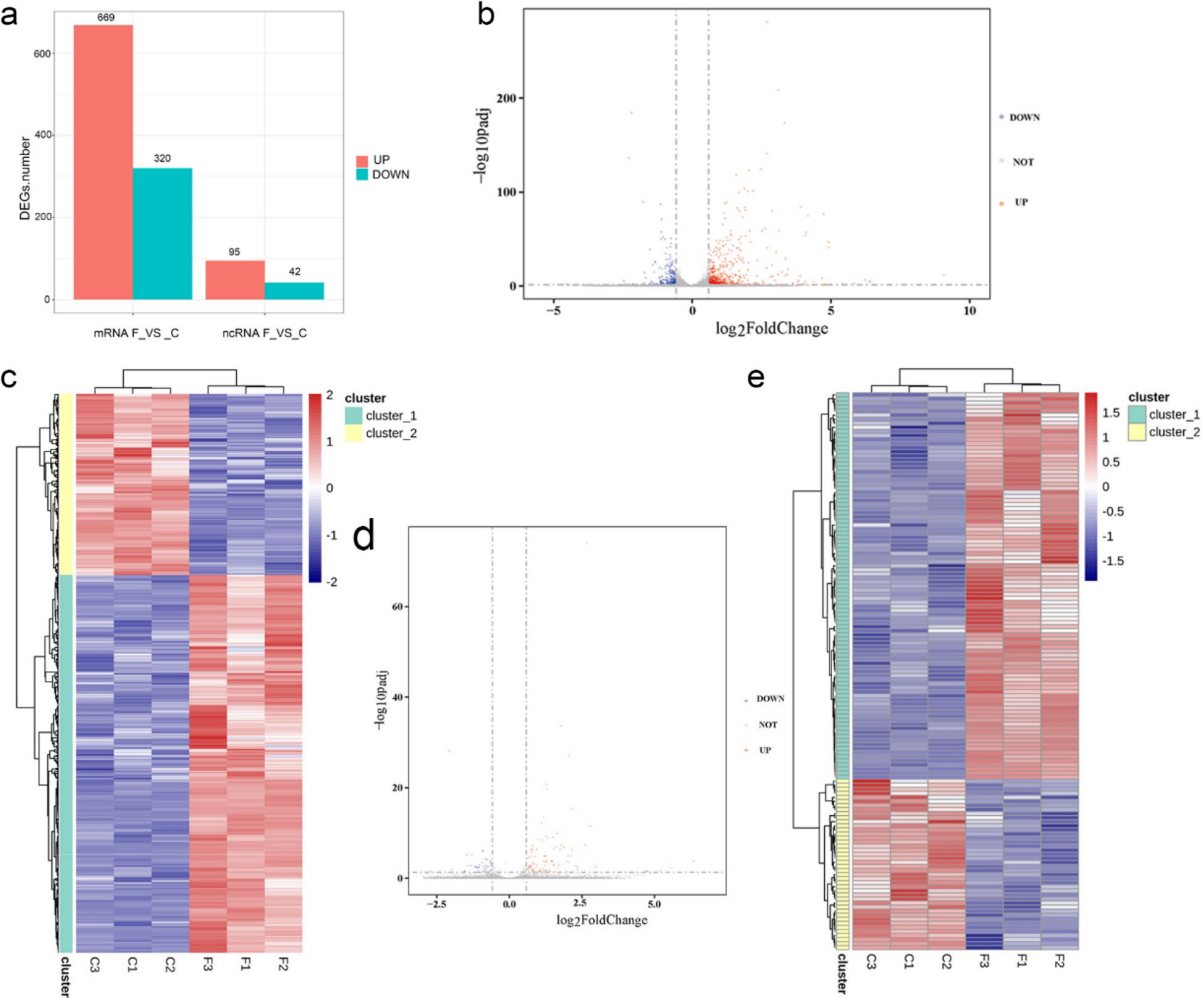
γ-T3 modifies the gene expression of mRNA and ncRNA in gastric cancer cells. **a** Differential expression analysis of mRNA and ncRNA genes. **b**, **c** Volcano and heat map analysis of mRNA in γ-T3-treated group and control group. **d**, **e** Volcano and heat map analysis of ncRNA in γ-T3-treated group and control group. Genes with |log_2_FoldChange|≥ log_2_1.5 and padj < 0.05 as differentially expressed genes screening criteria. F_VS_C: γ-T3-treated group VS control group. F: γ-T3-treated group; C: control group. *n* = 3

Next, we examined the expression of genes in gastric cancer cells by RNA-seq assay. The results showed that γ-T3 treatment altered the expression of genes in gastric cancer cells. We used Deseq2 for differential gene expression analysis to compare the treatment and control groups, and selected genes with |log_2_FoldChange|≥ log_2_1.5 and padj < 0.05 as differentially expressed genes screening criteria to obtain up- and down-regulated genes. There were 669 up-regulated genes and 320 down-regulated genes in mRNA in the γ-T3-treated group compared to the control group (Fig. 3a-c). In addition, there were 95 up-regulated genes and 42 down-regulated genes in ncRNA in the γ-T3-treated group compared to the control group (Fig. 3a, d-e). The findings suggest that γ-T3 affected gastric cancer cells and significantly altered the mRNA and ncRNA expression levels.

### γ-T3 effects gene function in gastric cancer cells

**Fig. 4:**
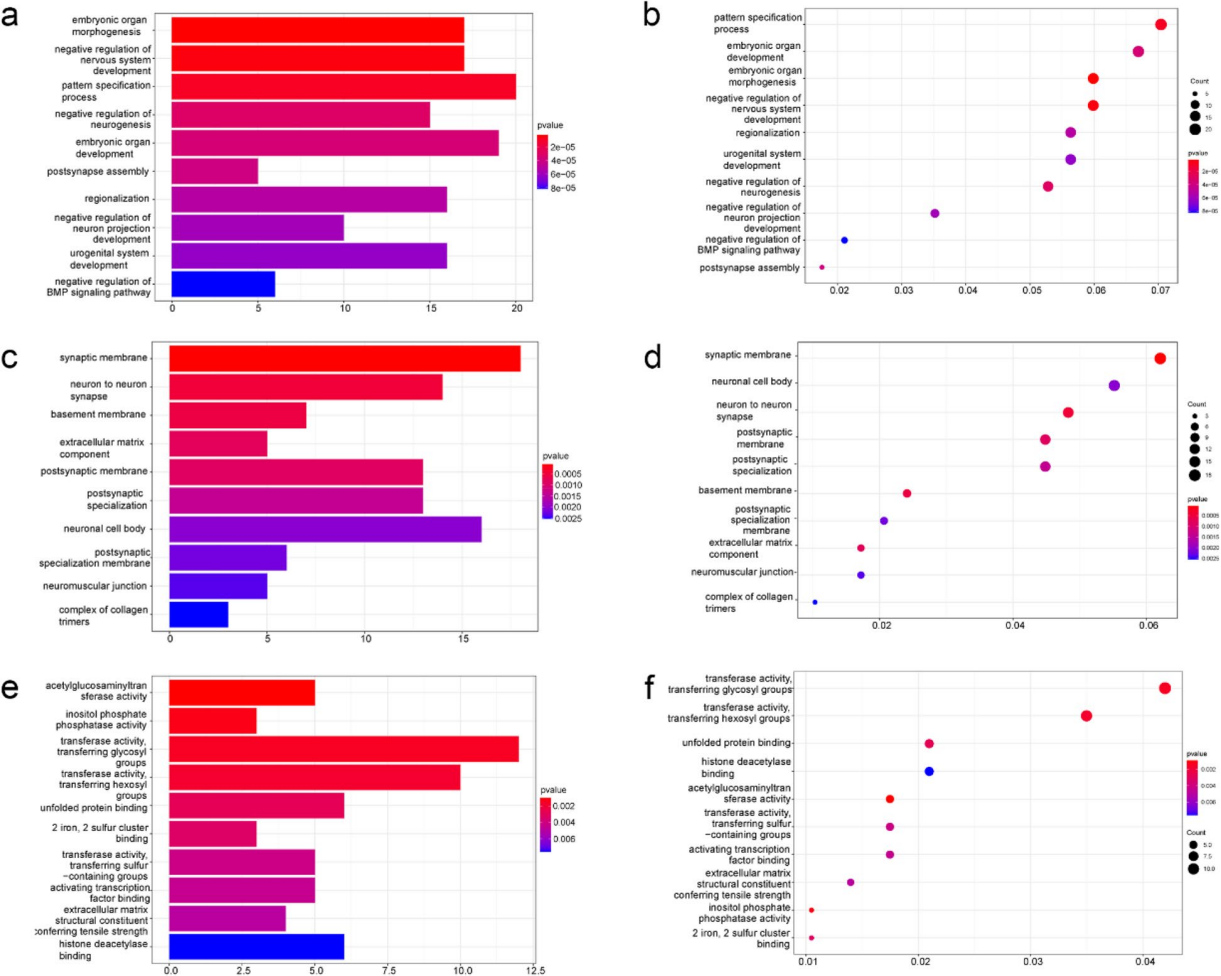
γ-T3 influences the gene function of gastric cancer cell. **a**, **b** BP involved in the decreased mRNA expression of genes in the γ-T3-treated group compared to the control group. **c**, **d** CC involved in the decreased mRNA expression of genes in the γ-T3-treated group compared to the control group. **e**, **f** MF involved in the decreased mRNA expression of genes in the γ-T3-treated group compared to the control group. *p* < 0.05. *n* = 3

We selected mRNAs with decreased gene expression after γ-T3 treatment of gastric cancer cells compared to control group for GO analysis. The biological process (BP), cellular component (CC), and molecular function (MF) of the genes that are down-regulated are summarized. The analysis revealed that γ-T3 treatment affected several gene MF of gastric cancer cells, including embryonic organ morphogenesis, negative regulation of nervous system development, pattern specification process, etc. (Fig. 4a, b). In addition, γ-T3 also significantly affected the CC of gastric cancer cells through synaptic membrane, neuron to neuron synapse, basement membrane, and extracellular matrix component (Fig. 4c, d). Furthermore, the analysis of BP revealed that γ-T3 significantly affected acetylglucosaminyltransferase activity, transferase activity, transferring glycosyl groups, unfolded protein binding, etc. in gastric cancer cells (Fig. 4e, f). These data indicate that γ-T3 plays a significant role in the molecular function, cellular component, and biological process of gastric cancer cell genes.

### γ-T3 induces apoptosis in gastric cancer cells

**Fig. 5:**
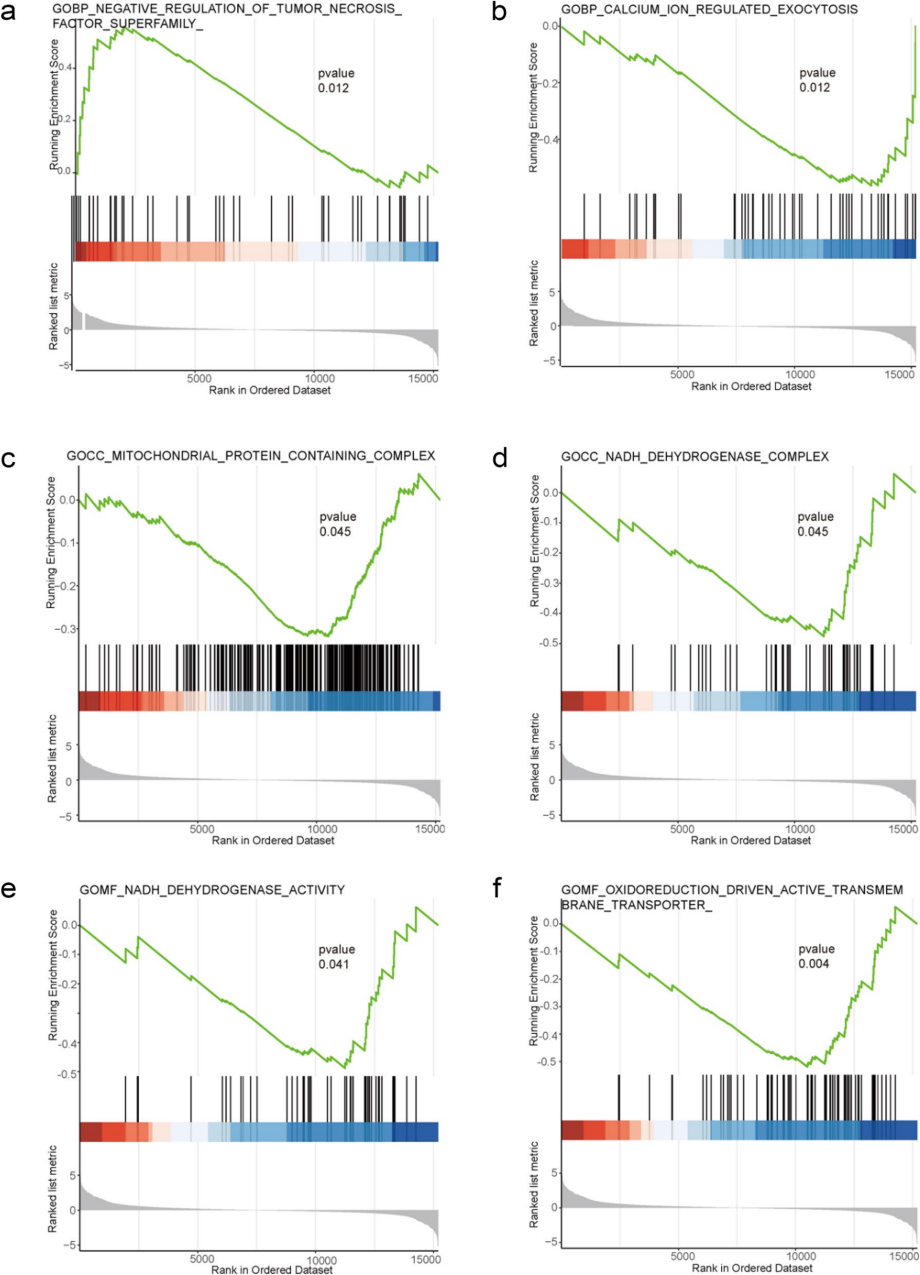
γ-T3 inhibits mitochondrial function and activates TNF signaling in gene function. **a**, **b** GSEA of γ-T3 in the BP of GO gene set. **c**, **d** GSEA of γ-T3 in the CC of GO gene set. **e**, **f** GSEA of γ-T3 in the MF of GO gene set. *p* < 0.05. *n* = 3

The sequencing data were analyzed for the treatment group compared to the control group. It was found that tumor necrosis factor (TNF) was able to kill cancer cells in mice, and the TNFR signaling pathway was able to induce apoptosis [10]. We performed GSEA analysis of the BP in GO and found that the pathway enriched to negatively regulate the TNF superfamily cytokines was activated (Fig. 5a). It is suggested that γ-T3 may induce apoptosis in gastric cancer cells through TNF signaling pathway. Calcium signaling functions extensively in many cellular processes and calcium signaling promotes the progression of several cancer types such as glioma [14, 33], prostate cancer [21] and breast cancer [32] by activating STAT3, an important transcription factor in cancer. Blocking calcium signaling may be a strategy to improve the anti-tumor immune response. The calcium-regulated extracellular secretory pathway was inhibited compared to the control group (Fig. 5b), suggesting that γ-T3 could act by downregulating the calcium signaling pathway.

Mitochondria are the main site of oxidative respiration in cells. The oxidative respiratory chain is composed of four protease complexes located on the inner mitochondrial membrane, called complex I (NADH dehydrogenase), complex II (succinate dehydrogenase), complex III ((ubiquinone-cytochrome chromogranin c reductase)) and complex IV (cytochrome c oxidase). Inhibition of tumor cell mitochondria may be an effective strategy for cancer therapy [61]. Furthermore, it was found that inhibition of NADH production in the cytoplasm significantly inhibited tumor growth [42]. Our previous study showed that γ-T3 induces apoptosis in gastric cancer cells through inhibition of mitochondrial complex I [59]. Analysis of the CC in GO revealed that the mitochondrial protein containing complex and NADH_dehydrogenase complex pathways were inhibited (Fig. 5c, d), which is consistent with our previous findings that γ-T3 inhibits the mitochondrial complex to induce apoptosis in gastric cancer cells.

In addition, MF analysis in GO also confirmed that γ-T3 inhibits NADH dehydrogenase activity and oxidoreduction-driven activity of active transmembrane transporters pathways acting in gastric cancer cells. (Fig. 5e, f). In summary, the analysis showed that γ-T3 induced apoptosis in gastric cancer cells through multiple pathways.

### γ-T3 inhibits oxidative phosphorylation

**Fig. 6:**
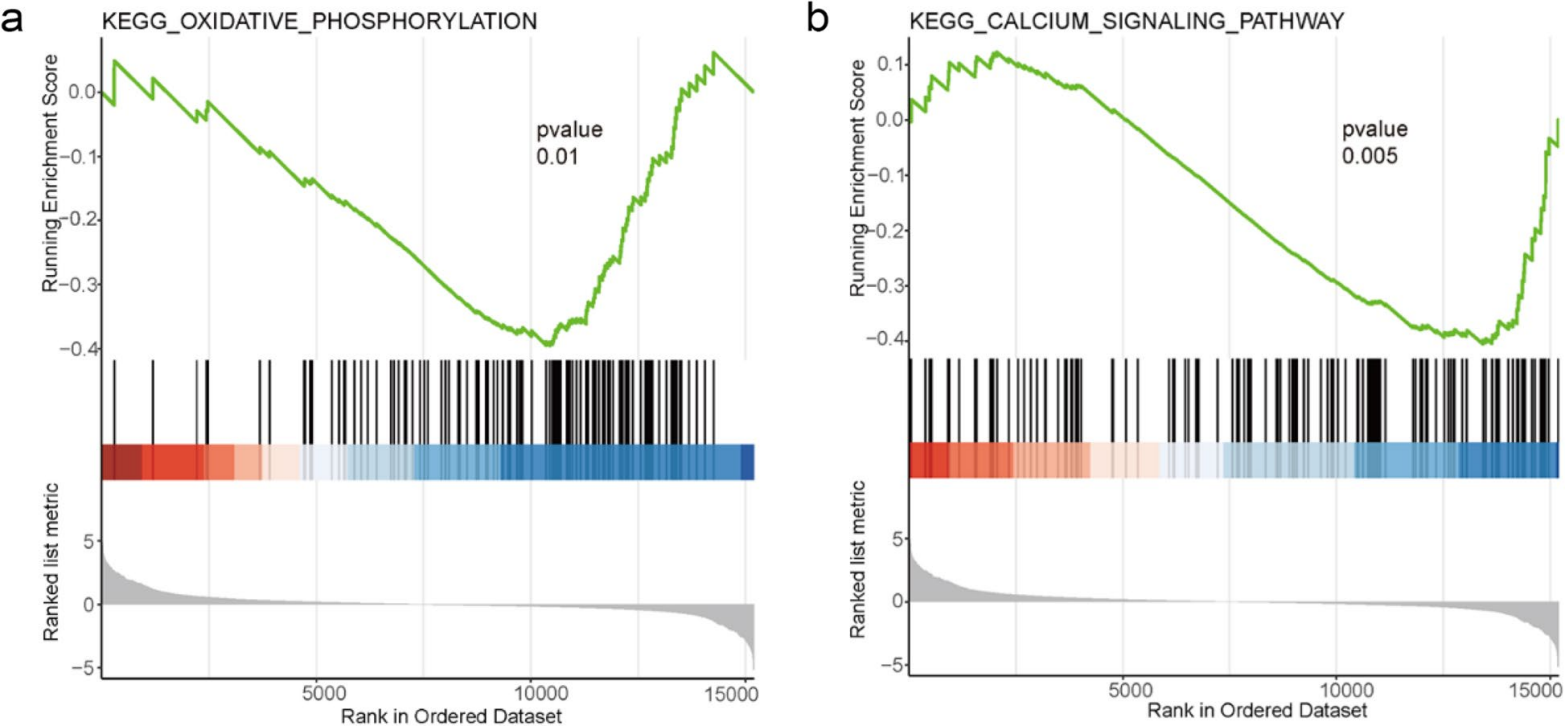
γ-T3 inhibits oxidative phosphorylation in gastric cancer cells. **a**, **b** GSEA of γ-T3 in the molecular function of KEGG gene set. *p* < 0.05. *n* = 3

Then, we performed GSEA on gene sets in KEGG. There is growing evidence that certain cancers are heavily dependent on oxidative phosphorylation (OXPHOS) and that OXPHOS inhibition is an effective means of targeting cancer [13, 58]. Our previous study also confirmed that γ-T3 treatment inhibited OXPHOS in gastric cancer cells [59]. The oxidative phosphorylation signaling pathway was inhibited in the treated group compared with the control group (Fig. 6a), which is consistent with our previous findings that γ-T3 can inhibit oxidative phosphorylation exerted to promote apoptosis in cancer cells. In a similar way, we found that the treated group inhibited the calcium signaling pathway compared to the control group (Fig. 6b), suggesting that γ-T3 acts on calcium signaling to inhibit cancer cells.

### γ-T3 triggers apoptosis by notch signaling pathway

**Fig. 7:**
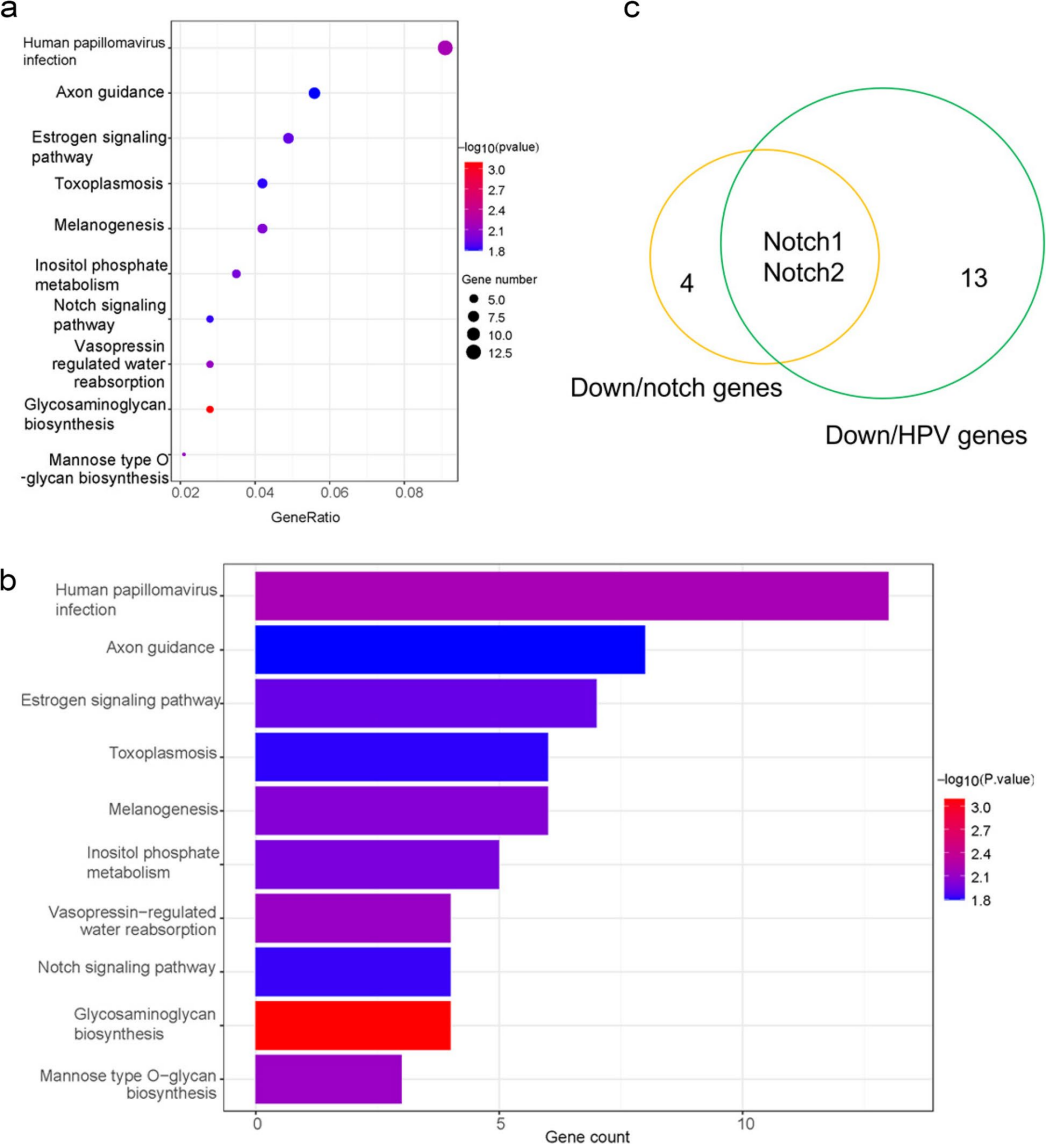
γ-T3 down-regulates notch1 and notch2 expression levels. **a**, **b** The KEGG pathway involved in the mRNA of the genes downregulated in the γ-T3 treatment group compared to the control group. **c** Venn diagram of genes involved in the HPV pathway and the notch signaling pathway in the γ-T3-treated group down-regulated pathway compared to the control group.* p* < 0.05. *n* = 3

We performed KEGG analysis of mRNA down-regulated in RNA-seq assay of γ-T3-treated gastric cancer cells. The analysis showed that genes downregulated in γ-T3-treated gastric cancer cells play significant roles in HPV pathway, melanogenesis, inositol phosphate metabolism, notch signaling pathway, etc. (Figs. 7a and 5b). Next, we found that HPV pathway intersects with the notch signaling pathway. Additionally, the genes involved in the two pathways were screened and the results showed that there were 13 down-regulated genes in the HPV pathway and 4 down-regulated genes in the notch signaling pathway. In particular, the HPV pathway and the notch signaling pathway have the same down-regulated genes, as notch1and notch2 (Fig. 7c). Studies have reported significantly elevated expression of notch1 and notch2 in gastric cancer tissues [23]. Our previous investigation identified that γ-T3 was able to induce apoptosis in gastric cancer cells [59]. Accordingly, the result proposed that γ-T3 may play a role in inducing apoptosis in gastric cancer cells by inhibiting the gene expression of notch1 and notch2.

## Discussion

T3 is a natural vitamin E derivative, and the anticancer properties of T3 were identified and studied [5, 47]. γ-T3 has been reported to inhibit the growth of a variety of human cancer cells in vivo and in vitro [1, 49, 56]. However, the mechanism of γ-T3 in gastric cancer research is not clear enough. Our previous study demonstrated that γ-T3 could induce apoptosis in gastric cancer cells [59]. Therefore, we performed RNA-seq assays on γ-T3-treated gastric cancer cells and untreated gastric cancer cells. There were 669 up-regulated genes and 320 down-regulated genes in mRNA in the γ-T3-treated group compared to the control group. In addition, there were 95 up-regulated genes and 42 down-regulated genes in ncRNA in the γ-T3-treated group compared to the control group. By KEGG analysis, we focused on the two pathways that ranked high in significant changes in γ-T3-treated gastric cancer cells, including HPV pathway and notch signaling pathway. We identified two common genes notch1 and notch2 in these two pathways. The expression levels of notch1 and notch2 were significantly downregulated in γ-T3-treated gastric cancer cells compared with the control group. The present results indicated that γ-T3 may play a role in inducing apoptosis in gastric cancer cells by down-regulating the expression levels of notch1 and notch2 through the notch signaling pathway.

With a large number of new cases each year, gastric cancer is a diagnosed malignancy worldwide. Gastric cancer is frequently diagnosed at an advanced stage and has a high mortality rate, making it a common cause of cancer-related deaths [53]. The treatment of gastric cancer remains a major challenge at this time. A major challenge is to translate the latest discoveries in molecular biology into effective treatments for patients with gastric cancer. The exploration of drug mechanisms in gastric cancer remains a major obstacle in the development of targeted therapeutics. Despite the current progress in the treatment of gastric cancer, poor prognosis and death still exist [7, 27, 28, 64].

Vitamin E has classic antioxidant properties. Natural vitamin E is a mixture of two compounds, tocopherols and T3 [41, 60]. Studies have reported that T3 have more significant neuroprotective and anticancer effects than tocopherols [31, 38, 45]. Several studies have found that T3 can induce apoptosis in a variety of cancer cells [3, 48, 54]. Furthermore, studies have reported a role for γ-T3 in inhibiting cancer cell proliferation [20, 50]. Our previous study showed that γ-T3 could induce apoptosis in gastric cancer cells and thus exert anti-cancer effects [59]. To further explore the specific mechanism by which γ-T3 exerts its anti-cancer effects, we treated gastric cancer cells with γ-T3 and performed RNA-seq assays on γ-T3-treated gastric cancer cells and untreated. The analysis revealed that consistent with our previous study, γ-T3 inhibited the mitochondrial complex and oxidative phosphorylation pathway and activated the TNF signaling pathway. This further confirmed the role of γ-T3 in treating gastric cancer cells.

Notch signaling is evolutionarily conserved [18]. Notch signaling is a cascade response that plays a key role in developmental processes, homeostasis and cell differentiation [8]. In mammals, notch signaling includes four receptors (notch1-4), and five ligands (Delta-like ligand-1, -3, -4 and jagged -1,2) [22]. Both receptors and ligands are transmembrane proteins and intercellular interactions induce signal transduction [66]. The notch signaling pathway regulates cell proliferation, cell cycle progression, differentiation and apoptosis [11, 19]. Notch-induced signaling has been reported to be associated with cancer progression, such as epithelial-to-mesenchymal transition and tumor development [6]. It has been demonstrated that the notch pathway plays a role in tumorigenesis [46]. It was found that activation of the notch pathway can promote the progression of gastric cancer [25, 43]. Moreover, notch signaling was found to be upregulated in gastric cancer, and gene expression of notch1, notch2, and notch3 was elevated in gastric cancer tissues [23, 24]. The mRNA expression levels of notch1 and notch2 were down-regulated in the γ-T3-treated group compared with the control group by analysis of RNA-seq assay. It is suggested that γ-T3 may exert anti-cancer effects through inhibiting notch signaling pathway.

To sum up, our previous study found that γ-T3 induced apoptosis in gastric cancer cells. The γ-T3-treated gastric cancer cells and the untreated group were subjected to RNA-seq assay. The results revealed that γ-T3 may play an anti-cancer role by inhibiting the notch signaling pathway through down-regulating the expression levels of notch1 and notch2. It is indicated that γ-T3 may become a new drug and a new therapeutic target for the treatment of gastric cancer by acting in the notch signaling pathway. To provide a basis for the clinical treatment of gastric cancer.

## Data Availability

Our data is not subject to ethical issues or other conflicts of interest.

## References

[1] Abdul Rahman Sazli F, Jubri Z, Abdul Rahman M, Karsani SA, Md Top AG, Wan Ngah WZ (2015). Gamma-tocotrienol treatment increased peroxiredoxin-4 expression in HepG2 liver cancer cell line. BMC Complement Altern Med.

[2] Abubakar IB, Loh HS (2016). A review on ethnobotany, pharmacology and phytochemistry of Tabernaemontana corymbosa. J Pharm Pharmacol.

[3] Agarwal MK, Agarwal ML, Athar M, Gupta S (2004). Tocotrienol-rich fraction of palm oil activates p53, modulates Bax/Bcl2 ratio and induces apoptosis independent of cell cycle association. Cell Cycle  (Georgetown, Tex.).

[4] Aggarwal BB, Sundaram C, Prasad S, Kannappan R (2010). Tocotrienols, the vitamin E of the 21st century: its potential against cancer and other chronic diseases. Biochem Pharmacol.

[5] Ahsan H, Ahad A, Iqbal J, Siddiqui WA (2014). Pharmacological potential of tocotrienols: a review. Nutr Metab (Lond).

[6] Allenspach EJ, Maillard I, Aster JC, Pear WS (2002). Notch signaling in cancer. Cancer Biol Ther.

[7] Aoyama T, Yoshikawa T (2017). Adjuvant therapy for locally advanced gastric cancer. Surg Today.

[8] Artavanis-Tsakonas S, Rand MD, Lake RJ (1999). Notch signaling: cell fate control and signal integration in development. Science.

[9] Ashburner M, Ball CA, Blake JA, Botstein D, Butler H, Cherry JM, Davis AP, Dolinski K, Dwight SS, Eppig JT, Harris MA, Hill DP, Issel-Tarver L, Kasarskis A, Lewis S, Matese JC, Richardson JE, Ringwald M, Rubin GM, Sherlock G (2000). Gene ontology: tool for the unification of biology. Gene Ontology Consortium Nat Genet.

[10] Ashkenazi A (2002). Targeting death and decoy receptors of the tumour-necrosis factor superfamily. Nat Rev Cancer.

[11] Bolos V, Grego-Bessa J, de la Pompa JL (2007). Notch signaling in development and cancer. Endocr Rev.

[12] Bray F, Laversanne M, Weiderpass E, Soerjomataram I (2021). The ever-increasing importance of cancer as a leading cause of premature death worldwide. Cancer.

[13] Caro P, Kishan AU, Norberg E, Stanley IA, Chapuy B, Ficarro SB, Polak K, Tondera D, Gounarides J, Yin H, Zhou F, Green MR, Chen L, Monti S, Marto JA, Shipp MA, Danial NN (2012). Metabolic signatures uncover distinct targets in molecular subsets of diffuse large B cell lymphoma. Cancer Cell.

[14] Chen L, Lin L, Xian N, Zheng Z (2019). Annexin A2 regulates glioma cell proliferation through the STAT3-cyclin D1 pathway. Oncol Rep.

[15] Chung FF-L, Tan PFTM, Raja VJ, Tan B-S, Lim K-H, Kam T-S, Hii L-W, Tan SH, See S-J, Tan Y-F, Wong L-Z, Yam WK, Mai CW, Bradshaw TD, Leong C-O (2017). Jerantinine A induces tumor-specific cell death through modulation of splicing factor 3b subunit 1 (SF3B1). Sci Rep.

[16] De Silva L, Chuah LH, Meganathan P, Fu JY (2016). Tocotrienol and cancer metastasis. BioFactors.

[17] Espinoza I, Miele L (2013). Notch inhibitors for cancer treatment. Pharmacol Ther.

[18] Gazave E, Lapebie P, Richards GS, Brunet F, Ereskovsky AV, Degnan BM, Borchiellini C, Vervoort M, Renard E (2009). Origin and evolution of the Notch signalling pathway: an overview from eukaryotic genomes. BMC Evol Biol.

[19] Giovannini C, Gramantieri L, Minguzzi M, Fornari F, Chieco P, Grazi GL, Bolondi L (2012). CDKN1C/P57 is regulated by the Notch target gene Hes1 and induces senescence in human hepatocellular carcinoma. Am J Pathol.

[20] Gysin R, Azzi A, Visarius T (2002). Gamma-tocopherol inhibits human cancer cell cycle progression and cell proliferation by down-regulation of cyclins. FASEB J.

[21] Henderson VM, Hawsawi O, Burton LJ, Campbell T, Trice K, Dougan J, Howard SM, Odero-Marah VA (2019). Cancer-bone microenvironmental interactions promotes STAT3 signaling. Mol Carcinog.

[22] Hori K, Sen A, Artavanis-Tsakonas S (2013). Notch signaling at a glance. J Cell Sci.

[23] Hu J, Yu J, Gan J, Song N, Shi L, Liu J, Zhang Z, Du J (2020). Notch1/2/3/4 are prognostic biomarker and correlated with immune infiltrates in gastric cancer. Aging (Albany NY).

[24] Huang T, Zhou Y, Cheng AS, Yu J, To KF, Kang W (2016). NOTCH receptors in gastric and other gastrointestinal cancers: oncogenes or tumor suppressors?. Mol Cancer.

[25] Jena N, Sheng J, Hu JK, Li W, Zhou W, Lee G, Tsichlis N, Pathak A, Brown N, Deshpande A, Luo C, Hu GF, Hinds PW, Van Etten RA, Hu MG (2016). CDK6-mediated repression of CD25 is required for induction and maintenance of Notch1-induced T-cell acute lymphoblastic leukemia. Leukemia.

[26] Joshi SS, Badgwell BD (2021). Current treatment and recent progress in gastric cancer. CA Cancer J Clin.

[27] Kanat O, O'Neil B, Shahda S (2015). Targeted therapy for advanced gastric cancer: a review of current status and future prospects. World J Gastrointest Oncol.

[28] Kanda M, Kodera Y, Sakamoto J (2015). Updated evidence on adjuvant treatments for gastric cancer. Expert Rev Gastroenterol Hepatol.

[29] Kanehisa M, Furumichi M, Tanabe M, Sato Y, Morishima K (2017). KEGG: new perspectives on genomes, pathways, diseases and drugs. Nucleic Acids Res.

[30] Kanehisa M, Goto S, Kawashima S, Okuno Y, Hattori M (2004). The KEGG resource for deciphering the genome. Nucleic Acids Res.

[31] Khanna S, Roy S, Ryu H, Bahadduri P, Swaan PW, Ratan RR, Sen CK (2003). Molecular basis of vitamin E action: tocotrienol modulates 12-lipoxygenase, a key mediator of glutamate-induced neurodegeneration. J Biol Chem.

[32] Li L, Zhang R, Liu Y, Zhang G (2020). ANXA4 Activates JAK-STAT3 Signaling by Interacting with ANXA1 in basal-like breast cancer. DNA Cell Biol.

[33] Li M, Ruan B, Wei J, Yang Q, Chen M, Ji M, Hou P (2020). ACYP2 contributes to malignant progression of glioma through promoting Ca efflux and subsequently activating c-Myc and STAT3 signals. J Exp Clin Cancer Res.

[34] Ling MT, Luk SU, Al-Ejeh F, Khanna KK (2012). Tocotrienol as a potential anticancer agent. Carcinogenesis.

[35] Love MI, Huber W, Anders S (2014). Moderated estimation of fold change and dispersion for RNA-seq data with DESeq2. Genome Biol.

[36] Mai C-W, Kang Y-B, Nadarajah VD, Hamzah AS, Pichika MR (2018). Drug-like dietary vanilloids induce anticancer activity through proliferation inhibition and regulation of bcl-related apoptotic proteins. Phytotherapy Research : PTR.

[37] Miyazawa T, Shibata A, Nakagawa K, Tsuzuki T (2008). Anti-angiogenic function of tocotrienol. Asia Pac J Clin Nutr.

[38] Nesaretnam K, Guthrie N, Chambers AF, Carroll KK (1995). Effect of tocotrienols on the growth of a human breast cancer cell line in culture. Lipids.

[39] Ntziachristos P, Lim JS, Sage J, Aifantis I (2014). From fly wings to targeted cancer therapies: a centennial for notch signaling. Cancer Cell.

[40] Omran AR (2005). The epidemiologic transition: a theory of the epidemiology of population change. 1971. Milbank Q.

[41] Packer L, Landvik S (1989). Vitamin E: introduction to biochemistry and health benefits. Ann N Y Acad Sci.

[42] Park J, Shim J-K, Kang JH, Choi J, Chang JH, Kim S-Y, Kang S-G (2018). Regulation of bioenergetics through dual inhibition of aldehyde dehydrogenase and mitochondrial complex I suppresses glioblastoma tumorspheres. Neuro Oncol.

[43] Piao HY, Guo S, Wang Y, Zhang J (2019). Long noncoding RNA NALT1-induced gastric cancer invasion and metastasis via NOTCH signaling pathway. World J Gastroenterol.

[44] Previs RA, Coleman RL, Harris AL, Sood AK (2015). Molecular pathways: translational and therapeutic implications of the Notch signaling pathway in cancer. Clin Cancer Res.

[45] Qureshi AA, Sami SA, Salser WA, Khan FA (2002). Dose-dependent suppression of serum cholesterol by tocotrienol-rich fraction (TRF25) of rice bran in hypercholesterolemic humans. Atherosclerosis.

[46] Revandkar A, Perciato ML, Toso A, Alajati A, Chen J, Gerber H, Dimitrov M, Rinaldi A, Delaleu N, Pasquini E, D'Antuono R, Pinton S, Losa M, Gnetti L, Arribas A, Fraering P, Bertoni F, Nepveu A, Alimonti A (2016). Inhibition of Notch pathway arrests PTEN-deficient advanced prostate cancer by triggering p27-driven cellular senescence. Nat Commun.

[47] Sailo BL, Banik K, Padmavathi G, Javadi M, Bordoloi D, Kunnumakkara AB (2018). Tocotrienols: The promising analogues of vitamin E for cancer therapeutics. Pharmacol Res.

[48] Sakai M, Okabe M, Tachibana H, Yamada K (2006). Apoptosis induction by gamma-tocotrienol in human hepatoma Hep3B cells. J Nutr Biochem.

[49] Sato C, Kaneko S, Sato A, Virgona N, Namiki K, Yano T (2017). Combination Effect of δ-Tocotrienol and γ-Tocopherol on Prostate Cancer Cell Growth. J Nutr Sci Vitaminol.

[50] Shah SJ, Sylvester PW (2005). Gamma-tocotrienol inhibits neoplastic mammary epithelial cell proliferation by decreasing Akt and nuclear factor kappaB activity. Exp Biol Med (Maywood, N.J.).

[51] Shen M, Chan TH, Dou QP (2012). Targeting tumor ubiquitin-proteasome pathway with polyphenols for chemosensitization. Anticancer Agents Med Chem.

[52] Shin-Kang S, Ramsauer VP, Lightner J, Chakraborty K, Stone W, Campbell S, Reddy SA, Krishnan K (2011). Tocotrienols inhibit AKT and ERK activation and suppress pancreatic cancer cell proliferation by suppressing the ErbB2 pathway. Free Radic Biol Med.

[53] Smyth EC, Nilsson M, Grabsch HI, van Grieken NC, Lordick F (2020). Gastric cancer. Lancet (London, England).

[54] Srivastava JK, Gupta S (2006). Tocotrienol-rich fraction of palm oil induces cell cycle arrest and apoptosis selectively in human prostate cancer cells. Biochem Biophys Res Commun.

[55] Sung H, Ferlay J, Siegel RL, Laversanne M, Soerjomataram I, Jemal A, Bray F (2021). Global Cancer Statistics 2020: GLOBOCAN Estimates of Incidence and Mortality Worldwide for 36 Cancers in 185 Countries. CA Cancer J Clin.

[56] Sylvester PW, Akl MR, Malaviya A, Parajuli P, Ananthula S, Tiwari RV, Ayoub NM (2014). Potential role of tocotrienols in the treatment and prevention of breast cancer. BioFactors.

[57] Tiwari RV, Parajuli P, Sylvester PW (2015). γ-Tocotrienol-induced endoplasmic reticulum stress and autophagy act concurrently to promote breast cancer cell death. Biochem Cell Biol.

[58] Vazquez F, Lim J-H, Chim H, Bhalla K, Girnun G, Pierce K, Clish CB, Granter SR, Widlund HR, Spiegelman BM, Puigserver P (2013). PGC1α expression defines a subset of human melanoma tumors with increased mitochondrial capacity and resistance to oxidative stress. Cancer Cell.

[59] Wang H, Luo J, Tian W, Yan W, Ge S, Zhang Y, Sun W (2019). γ-Tocotrienol inhibits oxidative phosphorylation and triggers apoptosis by inhibiting mitochondrial complex I subunit NDUFB8 and complex II subunit SDHB. Toxicology.

[60] Weber C, Podda M, Rallis M, Thiele JJ, Traber MG, Packer L (1997). Efficacy of topically applied tocopherols and tocotrienols in protection of murine skin from oxidative damage induced by UV-irradiation. Free Radical Biol Med.

[61] Wheaton WW, Weinberg SE, Hamanaka RB, Soberanes S, Sullivan LB, Anso E, Glasauer A, Dufour E, Mutlu GM, Budigner GS, Chandel NS (2014). Metformin inhibits mitochondrial complex I of cancer cells to reduce tumorigenesis. Elife.

[62] Wilankar C, Khan NM, Checker R, Sharma D, Patwardhan R, Gota V, Sandur SK, Devasagayam TPA (2011). γ-Tocotrienol induces apoptosis in human T cell lymphoma through activation of both intrinsic and extrinsic pathways. Curr Pharm Des.

[63] Wu WK, Cho CH, Lee CW, Fan D, Wu K, Yu J, Sung JJ (2010). Dysregulation of cellular signaling in gastric cancer. Cancer Lett.

[64] Yoo C, Park YS (2015). Companion diagnostics for the targeted therapy of gastric cancer. World J Gastroenterol.

[65] Yu G, Wang L-G, Han Y, He Q-Y (2012). clusterProfiler: an R package for comparing biological themes among gene clusters. OMICS.

[66] Yuan X, Wu H, Xu H, Xiong H, Chu Q, Yu S, Wu GS, Wu K (2015). Notch signaling: an emerging therapeutic target for cancer treatment. Cancer Lett.

